# Failure to thrive: Case definition & guidelines for data collection, analysis, and presentation of maternal immunisation safety data

**DOI:** 10.1016/j.vaccine.2017.01.051

**Published:** 2017-12-04

**Authors:** Elizabeth Ross, Flor M. Munoz, Bassey Edem, Cassandra Nan, Fyezah Jehan, Julie Quinn, Tamala Mallett Moore, Sanie Sesay, Hans Spiegel, Librada Fortuna, Sonali Kochhar, Jim Buttery, for The Brighton Collaboration Failure to Thrive Working Group

**Affiliations:** a Monash University, Department of Paediatrics, Monash Children’s Hospital, Melbourne, Australia; b Baylor College of Medicine, Departments of Paediatrics, Molecular Virology and Microbiology, Houston, TX, USA; c Vaccinology and Pharmaceutical Clinical Development, University of Siena, Italy; d GlaxoSmithKline, Stevenage, UK; e Aga Khan University, Department of Paediatrics and Child Health, Pakistan; f Monash Children’s Hospital, Infection and Immunity, Department of Paediatrics, The Ritchie Centre, Hudson Institute, Monash University, SAEFVIC, Murdoch Children’s Research Institute, Victoria, Australia; g Sanofi Pasteur Inc., Swiftwater, PA, USA; h Sanofi Pasteur, France; i Kelly Government Solutions (KGS), Rockville, MD, USA; j BioNet-Asia, Thailand; k Global Healthcare Consulting, India; l Monash University, Department of Paediatrics, Infection and Immunity, Monash Children’s Hospital, Monash Health; SAEFVIC, Murdoch Children’s Research Institute, Melbourne, Australia; m Erasmus University Medical Center, Rotterdam, The Netherlands

**Keywords:** Failure to thrive, Malnutrition, Adverse event, Immunisation, Guidelines, Case definition, FTT, Failure to thrive, HIC, High income countries, LMIC, Low and middle income countries, MUAC, Mid-upper arm circumference, SAM, Severe acute malnutrition, UNICEF, The United Nations Children's Fund, VAERS, Vaccine Adverse Event Reporting System, WHO, World Health Organisation

## 1 Preamble

### 1.1 Need for developing case definitions and guidelines for data collection, analysis, and presentation for failure to thrive as an adverse event following maternal immunisation

Failure to thrive (FTT) is a descriptive term for insufficient growth, usually identified in infancy. Definitions of FTT typically incorporate both clinical characteristics of insufficient growth and specific anthropometric criteria which define it.

Literature related to defining FTT can be broadly categorized in terms of anthropometric indices, aetiological causes, and a variety of variably interchangeable descriptors used to describe FTT or relevant clinical terms. There is no agreed definition and utilisation studies of existing definitions have largely been limited by small sample sizes, apart from two European infant cohorts. These studies agreed that all current means of diagnosing FTT either over or under diagnose the condition and a more accurate diagnostic tool could be beneficial [1], [2]. This paper focuses upon FTT in the first year of life, to assist the investigation of any potential concerns regarding reduced growth in infancy following antenatal maternal immunisation.

Key anthropometric terms include child growth, growth assessment, growth chart monitoring, growth monitoring, and nutritional status. Aetiological literature attempting to define FTT includes the areas of malnutrition and feeding disorders. Relevant clinical descriptors include child health, failure to thrive, growth interruption, growth retardation, weight/growth faltering, malnutrition, wasting, stunting and feeding disorder.

FTT is used as a diagnosis, or a description of a weight gain pattern.

Anthropometric indices have been used universally for defining FTT, yet there is heterogeneity regarding the specific indices, for example weight for age versus weight for height/length. Under-nutrition or inadequate nutrition is thought to be the underlying factor with the consequence of significant interruption to the expected rate of growth, and is strongly linked to malnutrition. In low income countries, malnutrition manifesting as failure to thrive is more common [3], [4], [5], [6], [7], [2].

**Normal growth** is described in reference to what is regarded as abnormal growth, falling below a pre-determined centile, usually the 3rd. This gives no direct estimate of growth, only of attained weight and clinically children may deviate from their earlier centile position. The growth curve of a normally growing child will represent gradual and incremental increases in weight from birth and is based on regular weight recordings [8], [9].

Among infants, determining FTT presents its own set of challenges. For example, in infants and young children with genetic short stature, prematurity, or intrauterine growth restriction who have an acceptable weight-for-length and normal growth velocity the term FTT is not used [10], [11]. Additionally, the well-recognized phenomenon of some healthy infants born above expected weight to experience an initial fall below birth centiles over the first 6–12 months, before following their “correct” centile, also known as ‘regression to the mean’, further complicates the specificity of FTT definitions based upon growth trajectory [12], [13]. This phenomenon has also been termed “catch down” growth [14].

Finally, there is little consensus as to what constitutes a normal rate of weight gain.

**Evolution of FTT as a description:**

The term FTT was not used until the 1930s and has evolved to describe under-nutrition resulting in a growth deviation or faltering regardless of the underlying cause. It is predominately based on anthropometrical parameters but no consensus on the choice of anthropometric indices has been agreed upon nor the criteria for abnormality. The literature acknowledges the lack of standardisation and recognizes that no one definition will be applicable or appropriate for all purposes but also acknowledges a need for clearly stated anthropometrical indices and cutoff points for the sake of scientific comparisons [3], [15], [16].

**Prevalence of FTT** depends on risks within populations. In low income settings, infectious diseases, poverty and inadequate nutrition are the primary risks.

In high income countries, the primary risks are preterm birth, and family dysfunction but in the majority of children with FTT an underlying medical condition is not found.

The described prevalence depends mainly on the definition being used and the demographics of the population being studied, with higher rates occurring in economically disadvantaged rural and urban areas. The UNICEF 2013 report states that globally, about one in four children under 5 years old are stunted from under-nutrition. An estimated 80 per cent of the world’s 165 million stunted children live in just 14 countries. Globally 26 per cent of children under 5 are stunted, however in sub-Saharan Africa and South Asia this number reaches nearer to 40 per cent, while in the most affected countries (Timor-Leste, Burundi, Niger and Madagascar) over 50 per cent of children under 5 are considered stunted [17] Studies show that in some developing countries growth faltering often starts soon after birth. In a study in Guatemala, depending on the reference standard used, 19 and 34% of the weight deficit observed in growth-restricted 3 year old children was attributed to failure to thrive during the first 3 months of life [18]. Children who are malnourished at 3–5 years of age often presented with anthropometric deficits at the end of their first year of life [19].

The community prevalence of FTT in high income countries is reported to be 1–10% under 2 years of age, with the United States seeing 5–10% of children presenting in primary care settings and 3–5% of children in hospital settings [7], [20], [21], [22]. In all settings there are a myriad of causes.

**Conventional classification of FTT:**

• Organic FTT-occurs in less than 5% of FTT cases-due to acute or chronic disorders that interfere with nutritional uptake such as cleft palate, malabsorption due to cystic fibrosis, and short gut syndrome.
• Non organic FTT-is attributed to inadequate intake with no apparent growth-inhibiting organic disorder. This can be due to environmental influences, stimulus deprivation-poverty, feeding techniques, or psychological reasons.
• Mixed FTT-organic and non-organic causes can overlap [23], [22], [24]

**Existing case definitions for failure to thrive:**

The difficulty in achieving a robust definition of FTT is due to both the limited number of available parameters to measure, and defining an abnormal change in these parameters given so much variability in normal patterns of growth in the first 12 months of life. While many infants can be expected to track along their defined growth centile, some healthy infants will not. Existing definitions of FTT do not easily distinguish between healthy infants with varied growth patterns and those who truly have FTT. A 2007 review of seven FTT criteria applied to a Danish birth cohort of 6090 infants found low specificity, with 27% of infants meeting one or more criteria, with most single criteria identifying less than half of the infants with known significant under-nutrition [2]. Healthy infants who may satisfy existing definitions of FTT include genetic short stature, preterm infants (if uncorrected for gestation), constitutional growth delay, and larger infants “regressing to the mean” [14].

FTT is consistently evaluated on anthropometrical indicators of weight and length/height. In practice, weight gain is the predominant indicator of choice. Weight is the first value affected in a child with FTT, followed by length if the FTT persists. A delay in growth of head circumference is only affected in severe cases, demonstrating the need to follow growth parameters over time. Assessment of length is desirable to better track stunting and to assess aetiology of low weight [7], [25], [26].

Using weight as the indicator FTT is described as a reduction in the expected rate of growth along an infant’s previously defined curve [27]. One common way of diagnosing FTT using weight is as a weight less than the third or fifth percentile for age on more than one occasion. This may falsely identify those infants naturally small, such as constitutional low weight while excluding larger infants with inadequate growth and true FTT whose weights do not fall below the lower centiles. An alternative definition of FTT is weight measurements that fall 2 major percentile lines between 2 or more time points. This definition may falsely include healthy infants who “regress toward the mean” while excluding infants with low weight where falling 2 major centiles is not possible [3], [4], [8], [21].

The measurement indices of weight and height/length in an equation to calculate a Body Mass Index (BMI) can also be used to identify children that are underweight. It is useful as a screening tool for wasting and thinness, but is not recommended for children younger than 2 years as there has been little research on what BMI calculated from length means in infancy [28], [29].

In low and middle income countries (LMIC) the use of body measurements are used to assess nutritional status. Body measurements in use include: weight; length in children under 24 months or under 87 cm in height; and mid-upper arm circumference (MUAC). Nutritional indices are calculated by comparing an individual’s measurements with that of a reference population. The nutritional indices commonly calculated for young children are:

• Weight for length: a measure of wasting or acute malnutrition.
• Height for age: a measure of stunting or chronic malnutrition.
• Weight for age: a measure of underweight or wasting and stunting combined.

However, as discussed earlier, faltering length presents later than changes in weight alone.

MUAC is also an important measure of wasting or acute malnutrition but is not an index by itself. It is used for rapid assessments as it identifies wasting and is a globally endorsed selection criteria for entry into selective feeding programs as it is a rapid and effective predictor of risk in children aged 6–59 months [30].

WHO and UNICEF recommend to define severe acute malnutrition (SAM) in infants using of weight for height indicators with the cutoff of below −3 standard deviations and a MUAC cut-off point of 115 mm to define SAM with MUAC [5], [31]. In infants aged from birth to 6 months, minimal data exists as to a reliable MUAC for diagnosing SAM. One small study from Indian suggests a lower range of 110 mm for infants 1–6 months of age [32].

The International Classification of Diseases (ICD-10) defines Failure to thrive as the lack of expected normal physiological development and defines Malnutrition as “The degree of malnutrition is usually measured in terms of weight, expressed in standard deviations from the mean of the relevant reference population. When one or more previous measurements are available, lack of weight gain in children, or evidence of weight loss in children or adults, is usually indicative of malnutrition. When only one measurement is available, the diagnosis is based on probabilities and is not definitive without other clinical or laboratory tests. In the exceptional circumstances that no measurement of weight is available, reliance should be placed on clinical evidence” [32].

As anthropometric indicators remain the measures of choice in low and high income communities, the working group elected to use weight-for-age as the primary measure. Weight is widely applicable with staff being skilled and most comfortable in measuring weight as a primary tool in all settings throughout LMIC and HIC. As previously discussed, length/height is not well validated in the target population of infants up to 12 months of age. Weight for age below the third or fifth centile does not adequately cover infants with FTT who do not fall to such low weights. As such weight for age with a fall through two major centiles is used as the primary diagnostic measure. Infants with FTT who score falsely negative in the upper levels of certainty may still be diagnosed with FTT based on a weight for length ratio below the third centile.

Accurate, reliable measurements are integral to growth monitoring and the clinical judgments of a child’s growth pattern. The choice of measuring equipment, implementation and interpretation of measurements, and the common practices embedded in many cultures present significant challenges to establishing robust definitions for FTT [33]. Correct measurement, plotting, and interpretation are essential for identifying growth problems. The measurements of weight, length/height and MUAC, in combination with the infant’s age and sex, gauge growth or failure to grow.

Age is generally recorded in months. This information can be derived from a known date of birth (such as from birth registration or health cards) or can be based on an estimate derived from a calendar of local events or mother’s recall [30], [34].

Various types of scales are used for weighing young children. The most precise form of measurement is using an electronic scale, widely used in HIC. These also allow the child to be measured in a care givers arms if necessary. In LMIC, the most commonly used in community settings is the hanging spring balance, which can weigh children up to 25 kg. Beam balance scales are also widely used in clinical settings, and are considered more precise than hanging spring balances.

Length is measured on a length board, infantometer, for a child less than 2 years old (less than 87 cm) in the recumbent position (crown-heel) measuring to the nearest 0.1 cm.

Growth charts are used to record a child’s measurement against children of the same age and sex, a reference standard. Centile charts are used and normal growth constitutes the tracking along the birth centile [5]. There are no percentile lines between 0 and 2 weeks of age as it is difficult to assess the normal postnatal weight loss. Signs indicative of poor weight gain for the neonate can include: a loss of more than 10% of birth weight in the first week, an average weight gain of less than 105 g per week or unexplained weight loss in infants 2 weeks to 3 months of age and poor weight gain compared to growth in length and head circumference [35].

In conjunction with growth charts, examination of clinical signs also determines a child who is failing to thrive. Physical examination can be used as an indicator for FTT where there are no measurements or are used in combination. Clinical signs include a loss of subcutaneous fat stores, poor muscle mass, loose skin folds, prominent ribs, thin limbs, sparse hair, rashes, pallor, lethargy, can indicate the level of FTT [23], [25], [36], [37], [38].

**Current assessment worldwide of FTT:**

The 2006 World Health Organisation (WHO) international growth charts for children <24 months are consistently recommended for use in the assessment of potential FTT. The WHO growth charts screen for possible abnormal or unhealthy growth. WHO recommends cutoff values of ±2 standard deviations, which correspond to the 2.3rd and 97.7th percentiles (modified to 2nd and 98th percentile) to define abnormal growth [39].

The charts illustrate the way healthy children should grow and are considered to be the gold standard for assessing the growth of young children [37], [40]. The growth of preterm infants (less than 37 weeks) can be monitored using the WHO Child Growth Standards with measurements plotted using corrected postnatal age for prematurity (ie, postnatal age in weeks − [40 weeks − gestational age in weeks]) until 24 or 36 months of age. Breastfed infants born with low birth weight will be expected to track along the lower percentiles of the WHO charts because exclusive breastfeeding does not change the fact that they were small for age at birth [41].

The desired outcome with the use of the WHO growth charts is to promote consistent practices in monitoring growth and assessing atypical patterns [40]

Indicators used to determine growth are: length/height-for-age; weight-for-age; weight-for-length/height.

**Limitations of growth charts:**

The basis of growth monitoring is measuring accurately, plotting on a growth chart and interpreting the growth curve. Growth data can therefore be inaccurate due to a number of factors including;

• deficiencies in the technical aspects of weighing and charting, and interpreting growth charts
• inadequate training of health workers
• situations where the child’s age cannot be accurately determined
• a child may also be underweight either because of short length/height (stunting) or thinness or both
• loss of follow-up, usually due to failure to attend clinics
• operational issues and maintenance of measurement equipment to ensure accuracy of measurements [42], [43]

**Failure to thrive and immunisation: What is known in literature?**

A search for terms describing FTT as adverse event following vaccination identified only articles suggesting immunisation visits as an opportunity for diagnosis of FTT, or under-immunisation being associated with increased risk of FTT [44], [45]. No articles were identified describing FTT as an adverse event following immunisation. A search of the US Vaccine Adverse Event Reporting System (VAERS) database (performed July 2016) described 47 reports where FTT was included in adverse event descriptions.

There is no uniformly accepted definition of FTT in vaccine pharmacovigilance. This represents a missed opportunity, as data comparability is crucial to enable both systematic analysis of clinical trial safety as well as meaningful surveillance and investigation of adverse events reported in existing programs. The increasing implementation of vaccination programs in pregnancy, and breadth of antenatal vaccine research in diverse healthcare settings where FTT is seen, makes the development of a definition able to be used in all of these settings urgent. The proposed case definition will refer only to infants up to 12 months of age.

### 1.2 Methods for the development of the case definition and guidelines for data collection, analysis, and presentation for failure to thrive as an adverse events following maternal immunisation

Following the process described in the overview paper [20] as well as on the Brighton Collaboration Website http://www.brightoncollaboration.org/internet/en/index/process.html, the Brighton Collaboration *Failure to Thrive Working Group* was formed in 2016 and included members of clinical, academic, public health and industry background. The composition of the working and reference group as well as results of the web-based survey completed by the reference group with subsequent discussions in the working group can be viewed at: http://www.brightoncollaboration.org/internet/en/index/working_groups.html.

To guide the decision-making for the case definition and guidelines, a literature search was performed using Medline, Embase and the Cochrane Libraries, including the terms infant, failure to thrive, growth retardation, growth failure, growth monitoring, growth faltering, under-nutrition and anthropometric. The search resulted in the identification of 1296 references, limited to English language. All abstracts were screened for possible reports of FTT following immunisation. One hundred and fifty articles with potentially relevant material were reviewed in more detail, in order to identify studies using case definitions or, in their absence, providing clinical descriptions of the case material. This review resulted in a detailed summary of 70 articles, including information on the study type, the vaccine, the diagnostic criteria or case definition put forth, the time interval since time of immunisation, and any other symptoms. Multiple general medical, paediatric and infectious disease text books were also searched.

Most publications identified were case series. The terminology was very inconsistent. Very few used case definitions at all and no two studies used the same definition. An inventory comprising nine relevant case definitions of FTT was made available to working group members.

### 1.3 Rationale for selected decisions about the case definition of failure to thrive as an adverse event following maternal immunisation

The term failure to thrive

– FTT is the most commonly used term to describe inadequate growth in infancy, however multiple terms exist, with aetiological descriptions often replacing FTT. The most common of these are malnutrition and under-nutrition, the most common causes of FTT worldwide.
– As with all Brighton Collaboration case definitions, the FTT definition presented includes differing levels. These do not reflect clinical severity, rather how confident FTT can be diagnosed from the information available about the individual case being assessed (see below). Given the wide range of settings, equipment used, measurements taken and number of assessment time points, this is especially important for FTT.

Related term(s) of Failure to Thrive

Malnutrition/under-nutrition represents the most common cause of FTT globally. Differential diagnoses of FTT that may present in infancy include: genetic short stature; constitutional growth delay; “regression to the mean” also known as “catch down growth”; and preterm infants whose gestational age is not corrected appropriately [14].

Formulating a case definition that reflects diagnostic certainty: Weighing specificity versus sensitivity

It needs to be re-emphasised that the grading of definition levels is entirely about diagnostic certainty, not clinical severity of an event. Thus, a clinically very severe event may appropriately be classified as Level 2 or 3 rather than Level 1 if it could reasonably be of non-FTT aetiology. Detailed information about the severity of the event should additionally always be recorded, as specified by the data collection guidelines.

The number of symptoms and/or signs that will be documented for each case may vary considerably. The case definition has been formulated such that the Level 1 definition is highly specific for the condition. As maximum specificity normally implies a loss of sensitivity, two additional diagnostic levels have been included in the definition, offering a stepwise increase of sensitivity from Level 1 down to Level 3, while retaining an acceptable level of specificity at all levels. In this way it is hoped that all possible cases of FTT can be captured.

Rationale for individual criteria or decisions made related to the case definition

It is widely agreed that a definition of FTT should be based on anthropometric data, although choice of which variables to use continues to be debated. Pathology, radiology and laboratory findings may have a role in determining the cause of failure to thrive and its differential diagnoses, but do not form part of this case definition.

Timing post maternal immunisation

Specific time frames for onset of symptoms following maternal immunisation are not included for the following main reasons:

The limited data available on possible FTT post maternal immunisation means a timeframe cannot be determined. For research purposes, the working group elected to focus the case definition on infants. That is, less than 12 month of age.

We postulate that a definition designed to be a suitable tool for testing causal relationships requires ascertainment of the outcome (e.g. FTT) independent from the exposure (e.g. immunisations). Therefore, to avoid selection bias, a restrictive time interval from immunisation to onset of FTT should not be an integral part of such a definition. Instead, where feasible, details of this interval should be assessed and reported as described in the data collection guidelines.

Further, FTT often occurs outside the controlled setting of a clinical trial or hospital. In some settings, it may be impossible to obtain a clear timeline of the event, particularly in less developed or rural settings. In order to avoid selecting against such cases, the Brighton Collaboration case definition avoids setting arbitrary time frames.

Differentiation from other (similar/associated) disorders

– Genetic short stature: infants born to small parents are typically small from birth and grow along their low percentile for both height and weight, sometimes dipping below their weight centile briefly. Parental height and weight information helps inform this diagnosis.
– Constitutional delay in growth: these infants may present with delayed length and occasionally weight compared with their same age peers. A family history of constitutional delay in parents or siblings may point to the diagnosis. Delayed bone age assessment is helpful above 12 months but is less reliable in infants.
– Preterm infants, if their growth is uncorrected on growth charts for gestation, will grow below the normal growth curve. Gestation of the baby in weeks should be recorded, and corrected before plotting on growth charts.
– Regression to the mean or “catch down growth” may occur in large babies (for example macrosomic babies born to mothers with gestational diabetes) may involve periods of lower growth that crosses centiles, followed by steady growth along a lower centile. Maternal history of diabetes and parental height and weight information may help identify this condition. However, this is a diagnosis made in retrospect typically, after the establishment of growth along the infant’s “true” centile.

FTT case definitions are limited by the difficulty in differentiating pathological FTT from the above conditions, especially in infancy when only limited time is available to observe growth patterns. Ideally, infants should be monitored regularly throughout their first 12 months to establish a true growth pattern and assess whether it is inadequate. Diagnosis of the above differentials should be based on history, examination and further investigations to exclude pathological causes of faltering growth.

Finally, pathological FTT results from a variety of causes which are not dealt with in these guidelines. These can be broadly grouped into one or more aetiological categories of poor oral nutrient intake, poor nutrient absorption, or increased caloric consumption (e.g. due to underlying disease). Similarly assessment of vaccine causality in FTT is not dealt with in this guideline, however should be managed in accordance with recent WHO vaccine adverse event guideline [46].

### 1.4 Guidelines for data collection, analysis and presentation

As mentioned in the overview paper, the case definition is accompanied by guidelines which are structured according to the steps of conducting a clinical trial, i.e. data collection, analysis and presentation. Neither case definition nor guidelines are intended to guide or establish criteria for management of ill infants, children, or adults. Both were developed to improve data comparability.

### 1.5 Periodic review

Similar to all Brighton Collaboration case definitions and guidelines, review of the definition with its guidelines is planned on a regular basis (i.e. every three to five years) or more often if needed. Validation of this case definition in developed and developing settings should help inform further revision of this case definition.

## 2 Case definition of failure to thrive^3^

**For all levels of diagnostic certainty**

FTT can be broadly defined as a faltering of growth from a previously established pattern of growth. It is universally established that a diagnosis of failure to thrive should be based on anthropometric data. However no consensus exits as to which measurements achieve the highest specificity and sensitivity. Weight is generally regarded is the indicator of choice, particularly a change in growth velocity, and as such has been selected as the standard for this case definition with a weight for age deceleration as the primary indicator of failure to thrive.

**Level 1 of diagnostic certainty**

– Infant age^4^ determined by a documented birth date
AND
– Weights obtained using an electronic scale
AND
– At least 2 weights, measured at least 4 weeks apart
AND
– Weight for age deceleration^5^ through at least 2 centile spaces on growth chart^6^

**Level 2 of diagnostic certainty**

**Level 2a:**

– Infant age determined by a documented birth date
AND
– Weights obtained using a beam balance scale
AND
– At least 2 weights, measured at least 4 weeks apart
AND
– Weight for age deceleration through at least 2 centile spaces on growth chart

*OR*

– Infants with an undocumented birth date, where age is determined based on Mothers recall to nearest month
AND
– Weights obtained using electronic scale
AND
– At least 2 weights, measured at least 4 weeks apart
AND
– Weight for age deceleration through at least 2 centile spaces on growth chart

**Level 2b:**

– Infant age determined by a documented birth date
AND
– Weights obtained using a spring balance scale
AND
– At least 2 weights, measured at least 4 weeks apart
AND
– Weight for age deceleration through at least 2 centile spaces on growth chart

*OR*

– Weight measured using electronic scale or beam balance scale
AND
– Length taken using Infantometer
AND
– Weight for length less than or equal to the 3rd centile on the appropriate growth chart

**Level 3 of diagnostic certainty**

**Level 3a:**

– Infants with an undocumented birth date, where age is determined based on Mothers recall to nearest month
AND
– Weight obtain using either beam balance or spring balance scale
AND
– At least 2 weights, measured as least 4 weeks apart
AND
– Weight for age deceleration through at least 2 centile spaces on growth chart

**Level 3b:**

– Infants with no weight available
AND
– Physical examination consistent with FTT^7^
AND
– MUAC^8^ indicative of severe wasting

## 3 Guidelines for data collection, analysis and presentation of failure to thrive

It was the consensus of the Brighton Collaboration *Failure to Thrive Working Group* to recommend the following guidelines to enable meaningful and standardised collection, analysis, and presentation of information about Failure to thrive. However, implementation of all guidelines might not be possible in all settings. The availability of information may vary depending upon resources, geographical region, and whether the source of information is a prospective clinical trial, a post-marketing surveillance or epidemiological study, or an individual report of Failure to Thrive. Also, as explained in more detail in the overview paper in this volume, these guidelines have been developed by this working group for guidance only, and are not to be considered a mandatory requirement for data collection, analysis, or presentation.

### 3.1 Data collection

These guidelines represent a desirable standard for the collection of data on availability following maternal immunisation to allow for comparability of data, and are recommended as an addition to data collected for the specific study question and setting. The guidelines are not intended to guide the primary reporting of Failure to Thrive to a surveillance system or study monitor. Investigators developing a data collection tool based on these data collection guidelines also need to refer to the criteria in the case definition, which are not repeated in these guidelines.

Guidelines numbers below have been developed to address data elements for the collection of adverse event information as specified in general drug safety guidelines by the International Conference on Harmonization of Technical Requirements for Registration of Pharmaceuticals for Human Use (ref), and the form for reporting of drug adverse events by the Council for International Organizations of Medical Sciences (ref). These data elements include an identifiable reporter and patient, one or more prior immunisations, and a detailed description of the adverse event, in this case, of Failure to Thrive following maternal immunisation. The additional guidelines have been developed as guidance for the collection of additional information to allow for a more comprehensive understanding of Failure to Thrive following maternal immunisation.

#### 3.1.1 Source of information/reporter

For all cases and/or all study participants, as appropriate, the following information should be recorded:

(1) Date of report.
(2) Name and contact information of person reporting^9^ and/or diagnosing the Failure to Thrive as specified by country-specific data protection law.
(3) Name and contact information of the investigator responsible for the subject, as applicable.
(4) Relation to the patient (e.g., immuniser [clinician, nurse], family member [indicate relationship], other).

#### 3.1.2 Vaccinee/control

##### 3.1.2.1 Demographics

For all cases and/or all study participants, as appropriate, the following information should be recorded:

(5) Case/study participant identifiers (e.g. first name initial followed by last name initial) or code (or in accordance with country-specific data protection laws).
(6) Date of birth, age, and sex.
(7) For infants: Gestational age and birth weight. Parental height and weight (both parents if known)

##### 3.1.2.2 Clinical and immunisation history

For all cases and/or all study participants, as appropriate, the following information should be recorded:

(8) Past medical history, including hospitalisations, underlying diseases/disorders, pre-immunisation signs and symptoms including identification of indicators for, or the absence of, a history of allergy to vaccines, vaccine components or medications; food allergy; allergic rhinitis; eczema; asthma. Maternal history of underlying diseases/disorders, gestational diabetes.
(9) Any medication history (other than treatment for the event described) prior to, during, and after immunisation including prescription and non-prescription medication as well as medication or treatment with long half-life or long term effect. (e.g. immunoglobulins, blood transfusion and immunosuppressants). Maternal medication history during pregnancy.
(10) Maternal immunisation history (i.e. previous immunisations and any adverse event following immunisation (AEFI)), in particular occurrence of Failure to Thrive after a previous immunisation.

#### 3.1.3 Details of the immunisation

For all cases and/or all study participants, as appropriate, the following information should be recorded for both infant and mother (if immunized in pregnancy):

(11) Date and time of immunisation(s).
(12) Description of vaccine(s) (name of vaccine, manufacturer, lot number, dose (e.g. 0.25 mL, 0.5 mL, etc) and number of dose if part of a series of immunisations against the same disease).
(13) The anatomical sites (including left or right side) of all immunisations (e.g. vaccine A in proximal left lateral thigh, vaccine B in left deltoid).
(14) Route and method of administration (e.g. intramuscular, intradermal, subcutaneous, and needle-free (including type and size), other injection devices).
(15) Needle length and gauge.

#### 3.1.4 The adverse event

(16) For all cases at any level of diagnostic certainty and for reported events with insufficient evidence, the criteria fulfilled to meet the case definition should be recorded.

Specifically document:

(17) Clinical description of signs and symptoms of Failure to Thrive, and if there was medical confirmation of the event (i.e. patient seen by physician).
(18) Date/time of onset^10^, first observation^11^ and diagnosis^12^, end of episode^13^ and final outcome.^14^
(19) Concurrent signs, symptoms, and diseases.
(20) Measurement/testing
  • Values and units of routinely measured parameters (e.g. temperature, blood pressure) – in particular those indicating the severity of the event;
  • Method of measurement (e.g. type of thermometer, oral or other route, duration of measurement, etc.);
  • Results of laboratory examinations, surgical and/or pathological findings and diagnoses if present.
(21) Treatment given for Failure to Thrive, especially specify what and dosing/duration if applicable.
(22) Outcome^12^ at last observation.
(23) Objective clinical evidence supporting classification of the event as “serious”.^15^
(24) Exposures other than the immunisation before and after immunisation (e.g. food, environmental) considered potentially relevant to the reported event.

#### 3.1.5 Miscellaneous/general

(25) The typical duration of surveillance for Failure to Thrive following antenatal vaccination is 12 months, however consideration should be given to:
  • Biologic characteristics of the vaccine e.g. live attenuated versus inactivated component vaccines;
  • Biologic characteristics of the vaccinee (e.g. nutrition, underlying disease resulting in immunosuppression.
(26) The duration of follow-up reported during the surveillance period should be predefined likewise. It should aim to continue to resolution of the event.
(27) Methods of data collection should be consistent within and between study groups, if applicable.
(28) Follow-up of cases should attempt to verify and complete the information collected as outlined in data collection guidelines 1 to 24.
(29) Investigators of patients with Failure to Thrive should provide guidance to reporters to optimise the quality and completeness of information provided.
(30) Reports of Failure to Thrive should be collected throughout the study period regardless of the time elapsed between immunisation and the adverse event. If this is not feasible due to the study design, the study periods during which safety data are being collected should be clearly defined.

### 3.2 Data analysis

The following guidelines represent a desirable standard for analysis of data on Failure to Thrive to allow for comparability of data, and are recommended as an addition to data analysed for the specific study question and setting.

(31) Reported events should be classified in one of the following five categories including the three levels of diagnostic certainty. Events that meet the case definition should be classified according to the levels of diagnostic certainty as specified in the case definition. Events that do not meet the case definition should be classified in the additional categories for analysis.

**Event classification in 5 categories**^16^

**Event meets case definition**

(1) Level 1: Criteria as specified in the Failure to Thrive case definition
(2) Level 2: Criteria as specified in the Failure to Thrive case definition
(3) Level 3: Criteria as specified in the Failure to Thrive case definition

**Event does not meet case definition**

***Additional categories for analysis***

(4) Reported Failure to Thrive with insufficient evidence to meet the case definition^17^
(5) Not a case of Failure to Thrive
(32) The interval between maternal immunisation and reported Failure to Thrive could be defined as the date/time of maternal immunisation to the date/time of onset^8^ of the first symptoms and/or signs consistent with the definition. If few cases are reported, the concrete time course could be analysed for each; for a large number of cases, data can be analysed in the following increments:

**Subjects with Failure to Thrive by Interval to Presentation**

**Table:** 

Interval∗	Number
<2 months after immunisation	
2–<6 months after immunisation	
6–<12 months after immunisation	
12–<18 months after immunisation	
Months increments thereafter	
Total

(33) The duration of a possible Failure to Thrive could be analysed as the interval between the date/time of onset^7^ of the first symptoms and/or signs consistent with the definition and the end of episode^11^ and/or final outcome^12^. Whatever start and ending are used, they should be used consistently within and across study groups.
(34) If more than one measurement of a particular criterion is taken and recorded, the value corresponding to the greatest magnitude of the adverse experience could be used as the basis for analysis. Analysis may also include other characteristics like qualitative patterns of criteria defining the event.
(35) The distribution of data (as numerator and denominator data) could be analysed in predefined increments (e.g. measured values, times), where applicable. Increments specified above should be used. When only a small number of cases is presented, the respective values or time course can be presented individually.
(36) Data on Failure to Thrive obtained from subjects receiving a vaccine should be compared with those obtained from an appropriately selected and documented control group(s) to assess background rates of hypersensitivity in non-exposed populations, and should be analysed by study arm and dose where possible, e.g. in prospective clinical trials.

### 3.3 Data presentation

These guidelines represent a desirable standard for the presentation and publication of data on Failure to Thrive following maternal immunisation to allow for comparability of data, and are recommended as an addition to data presented for the specific study question and setting. Additionally, it is recommended to refer to existing general guidelines for the presentation and publication of randomised controlled trials, systematic reviews, and meta-analyses of observational studies in epidemiology (e.g. statements of Consolidated Standards of Reporting Trials (CONSORT), of Improving the quality of reports of meta-analyses of randomised controlled trials (QUORUM), and of Meta-analysis Of Observational Studies in Epidemiology (MOOSE), respectively) [47].

(37) All reported events of Failure to Thrive should be presented according to the categories listed in guideline 31.
(38) Data on possible Failure to Thrive events should be presented in accordance with data collection guidelines 1–24 and data analysis guidelines 31–36.
(39) Terms to describe Failure to Thrive such as “low-grade”, “mild”, “moderate”, “high”, “severe” or “significant” are highly subjective, prone to wide interpretation, and should be avoided, unless clearly defined.
(40) Data should be presented with numerator and denominator (n/N) (and not only in percentages), if available.

Although immunisation safety surveillance systems denominator data are usually not readily available, attempts should be made to identify approximate denominators. The source of the denominator data should be reported and calculations of estimates be described (e.g. manufacturer data like total doses distributed, reporting through Ministry of Health, coverage/population based data, etc.).

(41) The incidence of cases in the study population should be presented and clearly identified as such in the text.
(42) If the distribution of data is skewed, median and range are usually the more appropriate statistical descriptors than a mean. However, the mean and standard deviation should also be provided.
(43) Any publication of data on Failure to Thrive should include a detailed description of the methods used for data collection and analysis as possible. It is essential to specify:
  • The study design;
  • The method, frequency and duration of monitoring for Failure to Thrive;
  • The trial profile, indicating participant flow during a study including drop-outs and withdrawals to indicate the size and nature of the respective groups under investigation;
  • The type of surveillance (e.g. passive or active surveillance);
  • The characteristics of the surveillance system (e.g. population served, mode of report solicitation);
  • The search strategy in surveillance databases;
  • Comparison group(s), if used for analysis;
  • The instrument of data collection (e.g. standardised questionnaire, diary card, report form);
  • Whether the day of immunisation was considered “day one” or “day zero” in the analysis;
  • Whether the date of onset^8^ and/or the date of first observation^9^ and/or the date of diagnosis^10^ was used for analysis; and
  • Use of this case definition for Failure to Thrive, in the abstract or methods section of a publication^18^^,^^19^.

## Disclaimer

The findings, opinions and assertions contained in this consensus document are those of the individual scientific professional members of the working group. They do not necessarily represent the official positions of each participant’s organisation (e.g., government, university, or corporation). Specifically, the findings and conclusions in this paper are those of the authors and do not necessarily represent the views of their respective institutions.

## Footnotes

3 The case definition should be applied when there is no clear alternative diagnosis for the reported event to account for the combination of symptoms.

4 **Age:** This case definition is limited to infants up to 12 months of age.

5 **Weight and Length:** Weight should be documented on the appropriate growth chart at the time of assessment. A fall through 2 centile spaces may be demonstrated at any point in the first 12 months of life, using any two weights as long as they are taken at least 4 weeks apart. Details of use of the weight balances allowable under this case definition and use of the Infantometer for length assessment are in Appendix A.

6 **Growth charts:** For infants born at 37 weeks gestation or above, the WHO growth charts should be applied. When using weight for age use the growth chart most accurate for the infants age. The birth to 6 months age range should be used where data is available for this range only, the birth to 2 years chart should be used where data is available beyond 6 months of life. When using weight for length, use the chart for birth to 2 years. For infants born less than 37 completed weeks gestation, the Intergrowth charts for postnatal growth standards in preterm infant should be used. All infants should be plotted on their respective growth chart using their corrected age. Links to relevant growth charts can be found in Appendix A.

7 Physical examination with signs of Failure to Thrive (must include at least 2 findings, with at least one major finding)Major findings: Reduced subcutaneous fat stores; poor muscle mass; loose skin folds; prominent ribs; thin limbsOther less specific signs include: sparse hair; rashes; pallor; miserable; lethargy/fatigue.

8 **Mid Upper Arm Circumference (MUAC):**For infants 0–6 months, a MUAC of ⩽ 110 mm is indicative of severe wasting.For infants 6–12 months, a MUAC of ⩽ 115 mm is indicative of severe wasting. Instructions on performing MUAC are in Appendix A.

9 If the reporting centre is different from the vaccinating centre, appropriate and timely communication of the adverse event should occur.

10 The date and/or time of onset is defined as the time post immunisation, when the first sign or symptom indicative for Failure to Thrive occurred. This may only be possible to determine in retrospect.

11 The date and/or time of first observation of the first sign or symptom indicative for Failure to Thrive can be used if date/time of onset is not known.

12 The date of diagnosis of an episode is the day post immunisation when the event met the case definition at any level.

13 The end of an episode is defined as the time the event no longer meets the case definition at the lowest level of the definition.

14 E.g. recovery to pre-immunisation health status, spontaneous resolution, therapeutic intervention, persistence of the event, sequelae, death.

15 An AEFI is defined as serious by international standards if it meets one or more of the following criteria: (1) it results in death, (2) is life-threatening, (3) it requires inpatient hospitalisation or results in prolongation of existing hospitalisation, (4) results in persistent or significant disability/incapacity, (5) is a congenital anomaly/birth defect, (6) is a medically important event or reaction.

16 To determine the appropriate category, the user should first establish, whether a reported event meets the criteria for the lowest applicable level of diagnostic certainty, e.g. Level three. If the lowest applicable level of diagnostic certainty of the definition is met, and there is evidence that the criteria of the next higher level of diagnostic certainty are met, the event should be classified in the next category. This approach should be continued until the highest level of diagnostic certainty for a given event could be determined. Major criteria can be used to satisfy the requirement of minor criteria. If the lowest level of the case definition is not met, it should be ruled out that any of the higher levels of diagnostic certainty are met and the event should be classified in additional categories four or five.

17 If the evidence available for an event is insufficient because information is missing, such an event should be categorised as “Reported Failure to Thrive with insufficient evidence to meet the case definition”.

18 An event does not meet the case definition if investigation reveals a negative finding of a necessary criterion (necessary condition) for diagnosis. Such an event should be rejected and classified as “Not a case of Failure to Thrive”.

19 Use of this document should preferably be referenced by referring to the respective link on the Brighton Collaboration website (http://www.brightoncollaboration.org).

## References

[1] Wright C.M. (1994). What is a normal rate of weight gain in infancy?. Acta Paediatr.

[2] Olsen E.M. (2007). Failure to thrive: the prevalence and concurrence of anthropometric criteria in a general infant population. Arch Dis Child.

[3] Olsen E.M. (2006). Failure to thrive: still a problem of definition. Clin Pediatr (Phila).

[4] Corbett S.S.D., Drewett R.F., Wright C.M. (1996). Does a fall down a centile chart matter? The growth and developmental sequelae of mild failure to thrive. Acta Paediatr.

[5] Shields B.W., Wacogne I., Wright C.M. (2012). Weight faltering and failure to thrive in infancy and early childhood. BMJ.

[6] Hughes I. (2007). Confusing terminology attempts to define the undefinable. Arch Dis Child.

[7] Government of Western Australia, D.o.H. Weight and growth issues in children; 2014. Available from: <http://www.pmh.health.wa.gov.au/general/CACH/docs/manual/3 Birth to School Entry/3.8/3.8.12.3 Weight_growth_background.pdf>.

[8] Wright C.M., Matthews J.N.S., Waterston A., Aynsley-Green A. (1994). What is a normal rate of weight gain in infancy?. Acta Paediatr.

[9] World Health Organization Global database on child growth and malnutrition. Geneva: WHO; 1997. <http://www.who.int/nutgrowthdb/en> [accessed Apr 16].

[10] Zenel J.A. (1997). Failure to thrive: a general pediatrician's perspective. Pediatr Rev.

[11] Bithoney W.G., Dubowitz H., Egan H. (1992). Failure to thrive/growth deficiency. Pediatr Rev.

[12] Cole T.J. (1997). 3-in-1 weight-monitoring chart. Lancet.

[13] Cole T.J. (1995). Conditional reference charts to assess weight gain in British infants. Arch Dis Child.

[14] Bergman P., Graham J. (2005). An approach to “failure to thrive”. Aust Fam Physician.

[15] Olsen E.M., Petersen J., Skovgaard A.M., Weile B., Jørgensen T., Wright C.M. (2007). Failure to thrive: the prevalence and concurrence of anthropometric criteria in a general infant population. Arch Dis Child.

[16] Peterson K.E., Chen L.C. (1990). Defining undernutrition for public health purposes in the United States. J Nutr.

[17] IMPROVING CHILD NUTRITION The achievable imperative for global progress. United Nations Children’s Fund (UNICEF); 2013 [cited 2016 3 March]. Available from: <http://www.unicef.org/gambia/Improving_Child_Nutrition_-_the_achievable_imperative_for_global_progress.pdf>.

[18] Rivera J., Ruel M.T. (1997). Growth retardation starts in the first three months of life among rural Guatemalan children. Eur J Clin Nutr.

[19] Shrimpton R. (2001). Worldwide timing of growth faltering: implications for nutritional interventions. Pediatrics.

[20] Cole S.Z., Lanham J.S. (2011). Failure to thrive: an update. Am Fam Physician.

[21] Rabinowitz SRG, Windle ML, Bhatia J. Nutritional considerations in failure to thrive; 2014.

[22] Spencer N.J. (2007). Failure to think about failure to thrive. Arch Dis Child.

[23] Habibzadeh H., Jafarizadeh H., Didarloo A. (2015). Determinants of failure to thrive (FTT) among infants aged 6–24 months: A case-control study. J Prev Med Hyg.

[24] Wright C.M. (1998). Effect of community based management in failure to thrive: randomised controlled trial. BMJ.

[25] Efron D. Failure to thrive, in: Isaacs PMSPD, editor, Practical paediatrics, 7th ed,. Elsevier; 2012. p. 102–105.

[26] Raynor P., Rudolf M.C. (2000). Anthropometric indices of failure to thrive. Arch Dis Child.

[27] McDonald E.L. (2008). Preventing growth faltering among Australian Indigenous children: implications for policy and practice. Med J Aust.

[28] Centers for disease control and prevention, National center for health statistics CDC growth charts. United States; 2009.

[29] Government of Western Australia, D.o.H. Growth monitoring. Conducting weight assessment in children 2–5 years; August 2014.

[30] United nations standing committee on nutrition: IASC global nutrition cluster’s harmonized training package (HTP); 2009.

[31] Becker P.J. (2014). Consensus statement of the Academy of Nutrition and Dietetics/American Society for Parenteral and Enteral Nutrition: indicators recommended for the identification and documentation of pediatric malnutrition (undernutrition). J Acad Nutr Diet.

[32] World health organization ICD-10: international statistical classification of diseases and related health problems, tenth revision; 2010.

[33] Experts’ consultation on growth monitoring and promotion strategies: program guidance for a way forward. New York, NY: United Nations Children's Fund; 2008. p. 8. [report of a technical consultation].

[34] WHO (1983). Measuring change in nutritional status.

[35] Tawia S., McGuire L. (2014). Early weight loss and weight gain in healthy, full-term, exclusively-breastfed infants. Breastfeed Rev.

[36] Lowen D., Jenny C. (2011). Failure to thrive. Child abuse and neglect: diagnosis, treatment and evidence.

[37] The WHO Child Growth Standards; 2006 [cited 2016 11 February]. Available from: <http://www.who.int/childgrowth/en/>.

[38] WHO, W.H.O. international statistical classification of diseases and related health problems ICD-10; 2016. p. 2.

[39] Grummer-Strawn LM, Reinold CM, Krebs NF. Use of World Health Organization and CDC growth charts for children aged 0–59 months in the United States. 20829749

[40] WHO Child Growth Standards. Length/height-for-age, weight-for-age, weight-for-length, weight-for-height and body mass index-for-age. Methods and development; 2006 [cited 2016 11 February ].

[41] A health professional’s guide for using the new WHO growth charts. Paediatr Child Health 2010;15(2):84–90. 10.1093/pch/15.2.84 PMC2865941 21286296

[42] Ashworth A., Shrimpton R., Jamil K. (2008). Growth monitoring and promotion: review of evidence of impact. Matern Child Nutr.

[43] UNICEF. Nutrition in emergencies; 2009.

[44] Msefula D. (1993). How can growth monitoring and special care of underweight children be improved in Zambia?. Trop Doct.

[45] Pedraza D.F., de Menezes T.N. (2014). Risk factors of stunting in preschool children: a case-control study. Cien Saude Colet.

[46] World Health Organisation. Causality assessment of adverse event following immunization (AEFI): user manual for the revised WHO classification; 2013 [cited 2016 October 20]. Available from: <http://www.who.int/vaccine_safety/publications/aefi_manual.pdf?ua=1>.

[47] Johansen M., Thomsen S.F. (2016). Guidelines for reporting medical research: a critical appraisal. Int Sch Res Notices.

