# Supplementary material for: Failure to thrive: Case definition & guidelines for data collection, analysis, and presentation of maternal immunisation safety data
Source: Vaccine. 2017 Dec 4;35(48Part A):6483–91. doi: 10.1016/j.vaccine.2017.01.051 (PMC5714432; doi:10.1016/j.vaccine.2017.01.051)
Supplement: Supplementary data 1 [file mmc1.docx]

# APPENDIX A: Tool to aid identification of appropriate level of diagnostic certainty

**Age:**

The working group decided on limiting the definition to infants up to 12 months of age. This is the most relevant age group in assessing effect of maternal vaccines during pregnancy and is consistent with pregnancy clinical trial guideline.

As such the case definition should be applied to infants up to 12 months of age. Birth date as a gold standard should be documented. Where no documentation exists, age can be determined based on a Mothers recall, to the nearest month. Where a mother cannot reliably recall the birth date, such infants may only be evaluated using low weight for length or clinical signs of wasting.

**Weight:**

Weight should be documented on the appropriate growth chart at the time of assessment. A fall through 2 centile spaces may be demonstrated at any point in the first 12 months of life, using any two weights as long as they are taken at least 4 weeks apart.

Weight measurements should be taken using:

Electronic scale- graduated to 10grams (Gold standard):

- Measures up to 20 kg in 0.01kg (10gm) increments
- Scale tared to zero grams
- Weight recorded in grams
- Scale calibrated twice a week
- Solidly built and durable
- Placed on level and hard surface
- Portable or fixed
- Motion detector and stabilizer
- weighed nude or in a clean diaper

Beam balance scale: graduate to 10 grams:

- Measures up to 20 kg in 0.01kg (10gm) increments
- Scale tared to zero grams
- Weight recorded in grams
- Scale calibrated twice a week
- Solidly built and durable
- Placed on level and hard surface
- Portable or fixed
- Motion detector and stabilizer
- weighed nude or in a clean diaper

Spring balance scale: child hangs in specially designed bag

- Measures up to maximum of 25kg
- Increments of 100g
- Scale needle tared to zero mark
- Accuracy checked before each measuring by comparing the scale reading with a known weight

**Length assessment using Infantometer**

- Range 30-110 mm with digital counter readings to 1 mm.
- calibrated twice a week
- crown-heel length, measures recumbent
- fixed head piece and sliding foot piece, head and feet flat against each
- placed on level hard surface, eye facing upward, fully extended
- measure without shoes and wearing light underclothing or diaper
- two trained individuals are required for an accurate measurement
- recorded to the last completed unit

**Growth charts:**

For infants born at 37 weeks gestation or above, the WHO growth charts should be applied. When using weight for age use the growth chart most accurate for the infants age. The birth to 6 months age range should be used where data is available for this range only, the birth to 2 years chart should be used where data is available beyond 6 months of life. When using weight for length, use the chart for birth to 2 years.

For infants born less than 37 completed weeks gestation, the Intergrowth charts for postnatal growth standards in preterm infant should be used.

All infants should be plotted on their respective growth chart using their corrected age.

Relevant growth charts can be found at:

Weight for age:

Birth to 6 months (girls) <http://www.who.int/childgrowth/standards/cht_wfa_girls_p_0_6.pdf?ua=1>

Birth to 6 months (boys) <http://www.who.int/childgrowth/standards/cht_wfa_boys_p_0_6.pdf?ua=1>

Birth to 2 years (girls) <http://www.who.int/childgrowth/standards/cht_wfa_girls_p_0_2.pdf?ua=1>

Birth to 2 years (boys) <http://www.who.int/childgrowth/standards/cht_wfa_boys_p_0_2.pdf?ua=1>

Weight for length:

Birth to 2 years (girls) <http://www.who.int/childgrowth/standards/cht_wfl_girls_p_0_2.pdf>

Birth to 2 years (boys) <http://www.who.int/childgrowth/standards/cht_wfl_boys_p_0_2.pdf?ua=1>

**Physical examination with signs of Failure to Thrive (must include at least 2 findings, with at least one major finding)**

Major findings:

- Reduced subcutaneous fat stores
- poor muscle mass
- loose skin folds
- prominent ribs
- thin limbs

Other less specific signs include:

- sparse hair
- rashes
- pallor
- miserable
- lethargy/fatigue

**Mid Upper Arm Circumference (MUAC):**

For infants 0-6 months, a MUAC of ≤110mm is indicative of severe wasting.

For infants 6-12 months, a MUAC of ≤115mm is indicative of severe wasting.

MUAC should be measured as follows:

- Use a non-stretchable measuring tape
- Measurement to be take at the midpoint of the upper arm
- Arm positioned by the infants side, palm facing upward
- Place a folded towel under the infants elbow to raise it slightly off the surface
- Measuring tape to be placed perpendicular to the long axis of the arm
- Tape should not cut into the skin
- Measure to the nearest mm
